# Broad applications of sensors based on laser-scribed graphene

**DOI:** 10.1038/s41377-023-01210-6

**Published:** 2023-07-05

**Authors:** Kuen Yao Lau, Jianrong Qiu

**Affiliations:** 1 Institute of Light+X Science and Technology, Faculty of Electrical Engineering and Computer Science, Ningbo University, 315211 Ningbo, Zhejiang, China; 2 State Key Laboratory of Modern Optical Instrumentation, College of Optical Science and Engineering, Zhejiang University, Hangzhou, 310027 China

**Keywords:** Graphene, Laser material processing

## Abstract

Sensors based on graphene materials have promising applications in the fields of biology, medicine and environment etc. A laser-scribed graphene provides a versatile, low-cost, and environmental friendly method for stress, bio, gas, temperature, humidity and multifunctional integrated sensors.

Real time detection of biological, chemical and physical information is important for medical diagnosis and environmental monitoring. Hence, it is urgent to develop high performance sensors with various functions and even multifunctions to meet the requirements of information era. Graphene is a unique material with two dimensional structure which has been proven to be an excellent material candidate for mechanical stress and strain, biochemical substances, as well as gases, temperature, and humidity sensors as illustrated in Fig. 1.

**Fig. 1:**
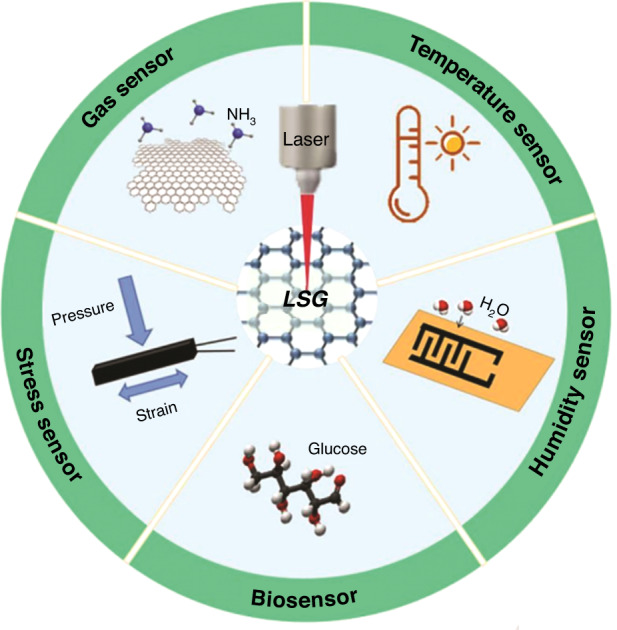
LSG for stress, bio, gas, temperature, and humidity sensors

To date, graphene has been fabricated through conventional methods such as mechanical exfoliation (ME), chemical vapor deposition (CVD), epitaxial growth (EG) and reduction of graphene oxide (rGO). The ME has low efficiency^1^, both CVD and EG requires high energy consumption and are expensive^2^, whereas rGO causes environmental pollution during preparation process^3^. Therefore, a method to produce graphene that avoids these drawbacks are of great interest. Laser direct writing (LDW) technique received great attention in recent years due to the advantages such as selective and localized reduction, precise and fast patterning, and the absence of masks and additional chemicals. The LDW technique was incorporated to irradiate carbon precursors and prepare graphene by in-situ scribing^4^. X. Liu and colleages from the University of Shanghai for Science and Technology and Queensland University of Technology presented a comprehensive review article entitled “Laser-Scribed Graphene for Sensors: Preparation, Modification, Applications and Future Prospects” on the existing laser-scribed graphene (LSG) for sensor fabrication in Light: Advanced Manufacturing^5^. In the review, a recent development of LSG in sensor applications was introduced and future prospects were provided.

The LSG could be prepared by two methods through the LDW: laser-reduced graphene oxide (GO)^6^ and laser-induced graphene^7^. For the first method, the LSG was prepared from the reduced GO using femtosecond laser irradiation. For the second method, the LSG was prepared by laser irradiation of polymer precursors such as polyimide and phenolic resin and natural materials such as paper and wood. The surface morphologies and properties of the LSG can be modified by adjusting laser parameters, controlling atmospheric condition and doping^8^. The laser parameters include laser power, scanning speed, and pulse repetition rate. For instance, a higher scanning speed and pulse repetition rate, and moisture atmosphere improve the surface hydrophilicity of the LSG. Doping improves the range and sensitivity of the LSG-based sensors. For instance, the creation of conductive channels between dopants and LSG increases the number of microcracks and resistance, which improves the performance of the stress and strain sensors, respectively.

Next, special attention was given to the application of LSG in various sensors in terms of strategies of designing sensors with high sensitivity, wide detection range, fast response time, and good repeatability. Stress sensor detects force signals by converting them into electrical signals. The LSG is mechanically strong, highly conductive and sensitive to various external applied stress, which changes the capacitance and resistance of the sensors^9,10^. Biosensors converts biological or biochemical reactions into physicochemical signals for analysis. The LSG contains electrocatalytic activity, strong adsorption capacity and sensitive to the detection of ions and biological molecules such as glucose, dopamine, hydrogen peroxide, nucleic acid and insulin^11,12^. The detection for changes in environment such as gas, temperature and humidity were discussed. The large number of three-dimensional micro-nanoporous structure in the LSG provides more active sites and diffusion paths for gas detection such as ammonia and hydrogen^13,14^. Temperature sensors were developed to real-time monitor a change in body temperature and battery temperature^15,16^. The LSG has high wearability and fast response time for a change between graphene resistance and temperature in a negative exponential relationship. A combination of LSG and GO shows higher sensitivity to humidity change than other functional conductive materials because the porosity of the LSG improves the hydrophilic nature of the GO to absorb water molecules^17^. Apart from typical sensors, multifunctional integrated sensors combine the detection of two or more physical or biological signals to enable more comprehensive and accurate applications in various fields such as a uric acid and tyrosine detection in sweat integrated with biochemical, temperature and strain sensors^18^.

Despite of the numerous advantages of the sensors integrated with the LSG, there exists several challenges for further development. For the preparation of LSG, investigations of new precursors, increase productivity, adhesion ability to biochemical substances and reduces defects are some worthwhile endeavors. To improve the performance of the sensors, designing suitable LSG surface microstructures and doping for stress sensors, employing electrochemical tests to analyze biosensors and incorporating ultrafine laser lithography to maximize the specific surface area of the LSG structures are recommended. We also expect that the LDW technique can be expanded to other materials and sensors with higher sensitivity, wider detection range, faster response time, and much better repeatability can be developed based on the deep understanding of laser matter interaction mechanism and development of precise control technique of laser induced structures.
